# Editorial: Reprogramming Stromal Cells in Chronic Inflammation and Cancer

**DOI:** 10.3389/fcell.2021.728439

**Published:** 2021-11-05

**Authors:** Ana Igea, Océane C. B. Martin, Tomer Cooks, Ioannis S. Pateras

**Affiliations:** ^1^ Biomedical Research Center (CINBIO), Universidade de Vigo, Vigo, Spain; ^2^ Universite de Bordeaux, CNRS, IBGC, UMR5095, Bordeaux, France; ^3^ The Shraga Segal Department of Microbiology, Immunology and Genetics, Ben-Gurion University of the Negev, Beer-Sheva, Israel; ^4^ Department of Histology and Embryology, Medical School, National and Kapodistrian University of Athens, Athens, Greece; ^5^ 2nd Department of Pathology, “Attikon” University Hospital, Medical School, National and Kapodistrian University of Athens, Athens, Greece

**Keywords:** inflammation, reprogramming, chronic inflammatory diseases, cancer, tumor microenvironment, 3D models, organoid, organotypic 3D culture

Despite extensive research effort in recent decades, the underlying mechanisms involved in the reprogramming of stromal cells in chronic inflammatory conditions and cancer are still ill-defined. This research topic includes original research and reviews of important concepts on inflammatory microenvironment remodeling and 3D models.

Adipose progenitor cells constitute a small and heterogeneous group of cells that can be modulated by mature adipocytes as well as by resident immune cells to determine their function and their differentiation potential. Several pathologies can destabilize the relationships created and break the equilibrium. In their review, Pyrina and others summarize the current knowledge about various factors and different immune and stromal cell populations that fate decisions of the adipose progenitor cells in the context of obesity-related inflammation. They define how in the course of obesity the pro-inflammatory microenvironment through multiple contact- and paracrine-mediated interactions shape the differentiation of preadipocytes, towards a pro-fibrotic transcriptional mode that in turn promotes fibrosis.

The NR4A receptors are potent sensors of changes in cellular microenvironment allowing the control of physiological and pathological processes. In their review, Murphy and Crean illustrate how this subfamily of nuclear receptors can be considered as transcriptional regulators in mesenchymal stromal cells, cell cycle progression and cytokine production to control local immune response in physiological or in chronic inflammatory conditions. Authors made a focus on recent advances in the pharmacological control of these receptors.

Gastrointestinal cancers are among the most frequent cancer-types worldwide and have been strongly linked with chronic inflammation. For that reason, current studies and therapies focus on cancer cells but also on the cells present in the tumor microenvironment (TME). Melissari and others review the role of cancer associated fibroblasts (CAFs), a major component of the TME, in tumor initiation, promotion and metastasis. They focus on the mechanisms that drive fibroblast reprogramming in gastrointestinal cancers, as well as on recent advances in therapies and patient prognosis.

The role of tumor infiltrating neutrophils in cancer progression of gastric cancer is highly appreciated (Jailon et al., 2020). Zhang and others demonstrated that tumor-educated neutrophils secreted IL-17, IL-23, and TNFα, that induced the transition of mesenchymal stem cells to CAFs. CAFs in turn promoted gastric cancer growth revealing a pro-tumorigenic role of neutrophils in cancer microenvironment.

In an additional publication focusing on CAFs, the emerging grasp of the TME is consistently unfolding even in cell-endogenous and evolutionally conserved mechanisms. In their research article, the group led by Ruth Scherz-Shouval have worked on the synchronized cellular circadian clock which was previously reported to facilitate a plethora of processes including gene regulation and homeostasis in the cell. The authors were able to reveal a novel aspect of the circadian clock disrupted during tumorigenesis and metastasis as cancer cells were compared to normal fibroblasts. Out of many related genes, Period 2 (Per2) was identified as a cardinal hub associated with this disruption. When Per2 was eliminated or knocked-out, the authors were able to abolish the metastatic progression. Notably, the expression of Per2 was found mainly in CAFs, a critical component of the TME.

The role of fibroblasts is further described in cutaneous T-cell lymphoma (CTCL) which refer to a group of lymphoproliferative diseases characterized by the accumulation of malignant T cells in chronically inflamed skin lesions. In their review, Stoleranco and others describe the crosstalk between fibroblasts, keratinocytes and malignant T cells and how they contribute to CTCL progression. Among the complex signaling networks, authors focus on the activation of STAT proteins, the development of a Th2 inflammatory microenvironment, neovascularization of the tumor tissue and changes in the skin architecture.

Notably, there is growing evidence describing the role of the TME in the development and progression of hepatocellular carcinoma (HCC) (Santhakumar et al., 2020). HCC typically arises from fibrotic or cirrhotic liver, characterized by alterations in extracellular matrix (ECM) components. Upon various non-tumoral components, such as immune and endothelial cells, the hepatic stellate cells (HSCs) are the tissue-specific pericytes that are now reported to play a major role in HCC progression. The article from the group of Hien Dang reviewed the recent findings connecting the dots in the hepatic TME promoted by intercellular interaction involving HSCs. As fibrosis and cirrhosis are the fundamental preceding conditions for HCC, activation of HSCs is a key factor that contributes to these processes. HSCs were reported to secrete pro-fibrogenic cytokines, to interact with neighboring liver natural killer (NK) cells, to induce senescence and to undergo an increase in DNA methylation, all contributing to their participation in supporting the tumor cells and their prosperity. Notably, HSCs were found to share intercellular cross talk with T-regulatory cells, Myeloid Derived Suppressor Cells (MDSCs) and endothelial cells suggested by their ability to produce angiogenic factors. Furthermore, HSCs were reported to interact with non-cellular elements in the tissue, mostly evident by the ability to remodel the ECM directly. The conception that HSCs play a dramatic role in HCC development, can be translated into the clinics by using drugs such as metformin (targeting the JNK pathway) or anti-angiogenic agents.

Two studies in this special issue highlight the advantages of 3D models to study human pathology. Vergnolle and others characterized morphological and functional phenotypes of colonic organoids from Inflammatory Bowel Disease (IBD) and control patients. The authors demonstrated that IBD organoid cultures preserved an inflammatory phenotype and that clinically used treatments reduced some parameters associated with this inflammatory phenotype. This study highlights that IBD patient organoids constitute a reliable human pre-clinical model to study IBD pathology and investigate new strategies targeting epithelial repair. In the second study, Haykal and others, depict the importance of using and developing new models and tools taking into consideration tissue complexity. A focus is made on organotypic models allowing to study cancer progression in complex settings and offering potential benefits for personalized medicine.

Altogether, these topical articles highlight the complex interactions between parenchymal cells and the surrounding stroma in different contexts. Besides, the advantages of 3D models to study the human pathophysiology of chronic inflammatory diseases and cancer and for pharmacological drug testing are underlined. In the era of personalized medicine, a profound knowledge of tissue microenvironment could improve prognosis and therapy in clinical practice.

## References

[B1] Jaillon S. Ponzetta A. Di Mitri D. Santoni A. Bonecchi R. Mantovani A. (2020). Neutrophil Diversity and Plasticity in Tumour Progression and Therapy. Nat. Rev. Cancer 20, 485–503. 10.1038/s41568-020-0281-y 32694624

[B2] Santhakumar C. Gane E. J. Liu K. McCaughan G. W. (2020). Current Perspectives on the Tumor Microenvironment in Hepatocellular Carcinoma. Hepatol. Int. 14 (6), 947–957. 10.1007/s12072-020-10104-3 33188512

